# Cavernous Hemangioma of the Sternocleidomastoid Muscle: A Case Report

**DOI:** 10.22038/IJORL.2023.67437.3302

**Published:** 2023-07

**Authors:** Maryam Akbari, Mir Mohammad Jalali, Fatemeh Azad

**Affiliations:** 1 *Otorhinolaryngology Research Center, Department of Otolaryngology and Head and Neck Surgery, School of Medicine, Guilan University of Medical Sciences, Amiralmomenin Hospital, Rasht, Iran.*

**Keywords:** Cavernous, Hemangioma, Neck muscles, Intramuscular, Sternocleidomastoid muscle

## Abstract

**Introduction:**

Cavernous hemangioma is a venous malformation and intramuscular hemangioma is a rare type of hemangioma. Most of these cases are reported in masseter and temporalis muscles, and the number of patients with hemangioma involving sternocleidomastoid (SCM) muscle is relatively less. The present study reported a case of intramuscular hemangioma and a literature review regarding hemangioma in the sternocleidomastoid muscle.

**Case Report:**

The present case was a 24-year-old woman with intramuscular hemangioma of the sternocleidomastoid muscle, manifesting a mass in the right supraclavicular region involving the sternocleidomastoid muscle. The woman was treated with surgery and achieved complete treatment. After surgery, the patient was kept under regular follow-up for the last six months without any evidence of recurrence.

**Conclusion:**

Intramuscular hemangioma of the sternocleidomastoid muscle is a rare entity that can present as a mass in the neck region. The treatment approach should be considered according to the diagnosis and site of vascular malformation.

## Introduction

Cavernous hemangioma is a venous malformation. The International Society for the Study of Vascular Anomalies (ISSVA) classifies cavernous hemangioma as a low-flow venous malformation (1). Cavernous hemangioma appears on the head and neck in 60 % to 70 % of cases (2). It can occur in any part of the body. Intramuscular hemangiomas are a rare type of hemangioma reported in less than 1% of all those (3). In the early stages, it may not cause obvious symptoms (4). In this study, we report intramuscular hemangioma of the sternocleido- mastoid muscle.

## Case report

A 24-year-old woman was referred to the ENT service with the presentation of a painless mass in the right supraclavicular region from 2 years ago. The mass had increased in size over that time. The patient had no history of trauma, surgery, inflammation, fever, nocturnal sweating, weight loss, dysphagia, shortness of breath, or hoarseness. Furthermore, there was no history of cutaneous neoplasms, visceral malignancies, or family members with similar presentation. In her past medical history, she had multiple sclerosis and is being treated with rituximab. 

A physical examination revealed a 3×4 cm firm, semi-mobile, semi-compressible, none tender, round mass in the right supraclavicular region. The skin over the swelling was normal and non-adherent to the underlying mass. 

**Fig 1:**
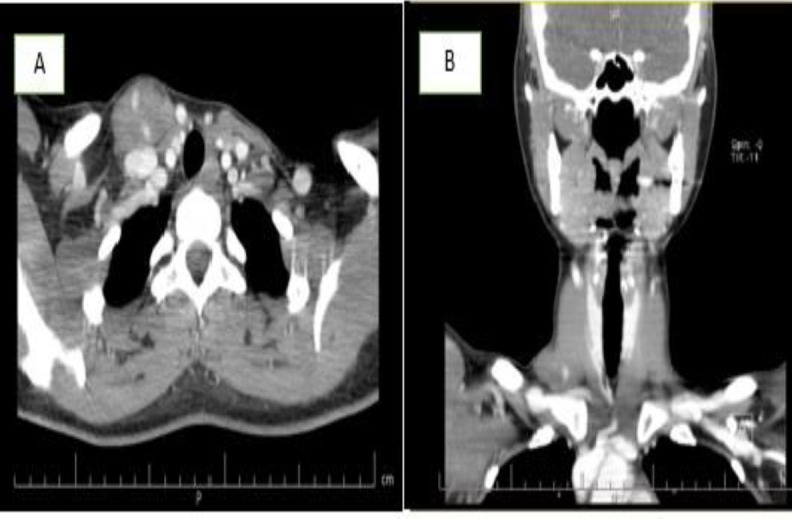
Axial (A) and coronal (B) CT images show heterogeneous enhancing solid mass measured 40×25×20 mm infiltrating the most distal part of the right sternocleidomastoid muscle in the base of the neck containing mottled hyperemic foci

**Fig 2:**
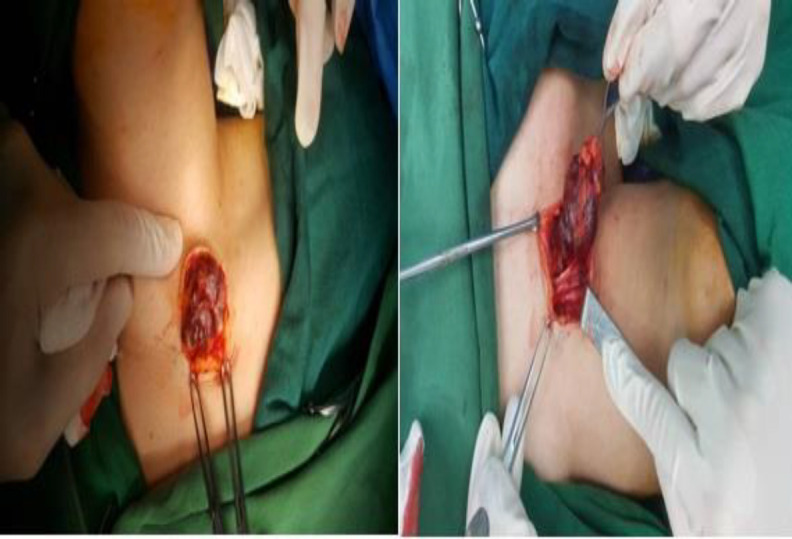
Intra-operative findings showed a partially encapsulated mass within the sternocleidomastoid muscle

There was no abnormal pulsation or bruit. Moreover, no cervical lymphadenopathy was appreciated. The CT scan of the neck with contrast revealed a heterogeneous solid mass measuring 40×25×20 mm infiltrated the most distal part of the right sternocleidomastoid muscle in the base of the neck containing mottled hyperemic foci. The CT scan report was compatible with mesenchymal sarcoma (Figure. 1). FNAC showed benign-looking follicular cells. Then ultrasonography of the neck was performed, with a normal thyroid on both sides. A heterogeneous focus contained multiple tubular, tortuous foci and low flow without connection to the right thyroid in the inferior-lateral region of the sternocleidomastoid muscle insertion on the right. The first diagnosis was vascular malformation. Doubting the FNA's answer, the FNA repeated, which was bloody. According to the findings, the decision was made to perform surgery. The surgical dissection showed a thrombosed vascular mass of the supraclavicular region originating in the sternocleidomastoid muscle (Figure. 2). 

The pedicle of the mass in the muscle was ligated, and the swelling was excised completely. Furthermore, there was no recurrence of the tumor at the 6-month follow-up. Histopathological examination confirmed the diagnosis of cavernous hemangioma.

**Fig 3:**
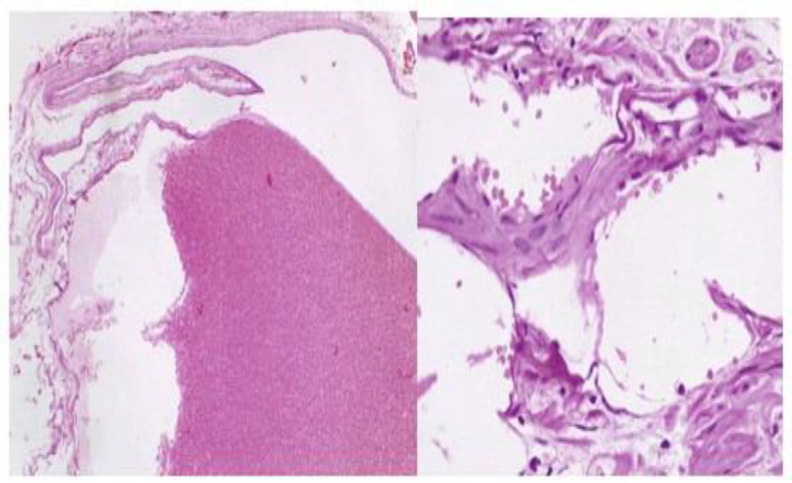
Microscopic inspection showed a benign vascular lesion, featuring cavernous hemangioma, composed of large dilated vascular channels lined by flat endothelium and RBC

A macroscopic inspection of the surgically excised specimen showed a well-defined mass lesion 4.3 × 3.2 × 2.5 cm, revealing a brownish-red solid cut surface with a soft consistency. Microscopic inspection showed a benign vascular lesion featuring cavernous hemangioma composed of large dilated vascular channels lined by flat endothelium (Figure. 3).

## Discussion

Numerous articles have reported intramuscular hemangioma in the head and neck regions. Most of these cases are reported in masseter and temporalis muscles, and the number of patients with hemangioma involving sternocleidomastoid (SCM) muscle is relatively less. As far as we searched, 12 cases of hemangioma with SCM muscle involvement have been reported in studies (3, 5-15). We reported a case of cavernous hemangioma of the SCM. Also, we add another case of cavernous hemangioma involving SCM muscle and review the literature for treatment and clinical aspects (Table 1). 

The present case in this study was female; in other studies, the prevalence of hemangioma in the SCM muscle was higher in females. According to published literature, it is more likely to occur on the right side than on the left side, and it was also present on the right side in our patient. The most common form in the studies was the cavernous type, similar to our study (Table 1).

**Table 1:** Summary of cases with cavernous and capillary hemangioma of the sternocleidomastoid muscle in previous studies

**Author/ Year**	**Sex (M/F)/Age (year)**	**Side**	**Duration of clinical history(month/ year)**	**Type of hemangioma**	**Treatment**
Wolf et al.,1985 (5)	F/ 30	right	5 y	cavernous	surgery
Broniatowski et al., 1993 (6)	M/ 27	left	3 m	cavernous	surgery
Chaudhary et al., 1998 (7)	M/15	right	1/5 y	cavernous	surgery
Lee et al., 2005 (3)	M/42	left	Not mentioned	capillary	surgery
Ferri et al., 2007 (8)	F/32	right	6 m	cavernous	surgery
Moumoulidis et al., 2007 (9)	F/71	right	2 y	cavernous	surgery
Pistor et al., 2011 (10)	Not mentioned / 52	left	5 y	cavernous	surgery
Bal et al., 2012 (11)	F/14	right	1 y	Not mentioned	surgery
Lad et al., 2017 (12)	F/ 28	right	2 y	cavernous	surgery
Biswas et al., 2017 (13)	M/ 10	left	after birth	cavernous	surgery
Kim et al., 2018 (14)	M/ 32	left	2 m	capillary	surgery
Sigdel et al., 2020 (15)	F/6	right	4 y	cavernous	surgery

According to the ISSVA (International Society for the Study of Vascular Anomalies) classification, Cavernous hemangioma is a slow-flow venous malformation (1). Another classification of intramuscular hemangioma is based on the size of the vessel and histologic features, which includes three groups: cavernous (large vessels, >140 mm), capillary (small vessels, ˂140 mm), and mixed. Capillary type is higher than cavernous type, so capillary type constitutes 50% of all intramuscular hemangiomas and 68% of head and neck intramuscular hemangiomas. The incidence of cavernous hemangioma of the head and neck is 26%; the recurrence rate of capillary type is 20 %, but recurrence of cavernous hemangioma is reported at 9% (8). The pathogenesis of hemangioma is unknown (1). According to previous studies, hemangioma is probably to be caused by abnormal embryonic vascular proliferation (3). 

The venous malformation can occur in any part of the body. Sixty to seventy percent of cavernous hemangioma occurs on the head and neck (2). The incidence of hemangioma in one-year-old children is 5%-10%. They occur more often in women than men (ratio: 3:1–5:1) and are more common in the second and third decades (16). 

Intramuscular hemangioma is a rare type of hemangioma that is reported in less than 1% of all. The masseter muscle is the most commonly reported hemangioma. Other muscles in which hemangioma has been reported include the trapezius, temporalis, orbital muscles, and SCM (3). Cavernous hemangioma has also been reported in large cervical vessels, such as the carotid sheet, and internal and external jugular veins (17). Venous malformation in the early stages may not cause obvious symptoms, but in advanced stages, depending on the location of the mass, there may be symptoms such as pain, pulsation, bruits, or compressive effects on neighboring organs (8). One symptom highly suggestive of vascular masses in the present case is that the mass size increases with physical activity. In addition to history and physical examination, paraclinical methods help diagnose vascular masses. Ultrasound (us) can help determine the vascular nature of the mass. Doppler is useful for identifying high and low-flow vascular malformation. MRI is the best method for evaluating vascular malformations and their association with surrounding structures. MRA is useful for assessing low and high-flow venous malformation and anatomical detail. CT scan is useful for revealing soft tissue tumors but cannot differentiate between the mass, muscles, or surrounding structures (18). Differential diagnoses of intramuscular hemangiomas include lymphadenopathy, salivary gland, muscular neoplasms, lymphangioma, and thyroid lesions (8). Several treatment methods for venous malformation include corticosteroid treatment, embolization, sclerotherapy، surgery, and laser therapy or combined therapy. Recent studies show that low-flow venous malformation treatment is more commonly endovascular sclerosants. As endovascular sclerosants, sodium tetradecyl sulfate (STS) is more widely used than Ethanol due to lower complication rates. However, in most articles, complete surgical excision is the method to treat cavernous hemangioma. Recurrence of cavernous hemangioma after surgery was reported as 20% (19-20).

## Conclusion

The present study reported a female of intramuscular hemangioma in the sternocleidomastoid muscle. Intramuscular hemangioma is a rare entity that can present as a mass in the neck region. The treatment approach should be considered according to the diagnosis and site of vascular malformation.
